# Synchronization of *E. coli* Bacteria Moving in Coupled Microwells

**DOI:** 10.1002/smll.202407832

**Published:** 2024-11-25

**Authors:** Aleksandre Japaridze, Victor Struijk, Kushal Swamy, Ireneusz Rosłoń, Oriel Shoshani, Cees Dekker, Farbod Alijani

**Affiliations:** ^1^ Delft University of Technology Delft 2628 CD The Netherlands; ^2^ Ben‐Gurion University of the Negev Beer‐Sheva 841050 Israel

**Keywords:** microswimmers, microcavities, synchronization

## Abstract

Synchronization plays a crucial role in the dynamics of living organisms. Uncovering the mechanism behind it requires an understanding of individual biological oscillators and the coupling forces between them. Here, a single‐cell assay is developed that studies rhythmic behavior in the motility of *E. coli* cells that can be mutually synchronized. Circular microcavities are used to isolate *E. coli* cells that swim along the cavity wall, resulting in self‐sustained oscillations. Connecting these cavities by microchannels yields synchronization patterns with phase slips. It is demonstrated that the coordinated movement observed in coupled *E. coli* oscillators follows mathematical rules of synchronization which is used to quantify the coupling strength. These findings advance the understanding of motility in confinement, and open up new opportunities for engineering networks of coupled oscillators in microbial active matter.

## Graphical abstract

Synchronization plays a crucial role in biology and physics, across different time and length scales. Here, the discovery of hydrodynamic synchronization amongst one of the smallest living organisms, *E. coli* bacteria, is reported, wheir their random motion is tamed and their swimming patterns are synced by using coupling microcavities.

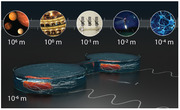

## 1 Introduction

Life at low Reynolds numbers remains intriguing.^[^ ^1^ ^]^ Flagellum‐driven motility enables bacterial cells to explore their environment, find nutrients, and avoid toxins.^[^ ^2^, ^3^ ^]^ Bacterial motility also provides insights into the formation of biofilms,^[^ ^4^ ^]^ bacterial swarming,^[^ ^5^ ^]^ rheotaxis,^[^ ^6^ ^]^ the propagation of infections,^[^ ^7^ ^]^ and has been even used as a measure to determine the efficacy of antibiotics in rapid antibiotic susceptibility testing.^[^ ^8^, ^9^ ^]^

It is well‐established that motile bacteria such as *Escherichia coli* exhibit a random‐walk behavior,^[^ ^10^, ^11^ ^]^ with periods of straight locomotion (swimming phase) that alternate with moments of abrupt reorientation (tumbling phase). Near flat surfaces, however, *E. coli* cells suppress their tumbling frequency^[^ ^12^ ^]^ and follow a circular trajectory.^[^ ^13^, ^14^ ^]^ This sporadic periodic movement is rooted in the spatial distribution of the flagella bundle and hydrodynamic interaction with the nearby surface. Interestingly, it has also been demonstrated that these rotations can get weakly phase‐locked to one another in dense populations and lead to large‐scale collective dynamics, for which the origins have remained incompletely understood.^[^ ^15^ ^]^ It is of interest to understand the microscopic processes behind this coordinated movement and devise effective strategies to control it.

Here, we engineer single‐cell *E. coli* clocks and develop an assay to systematically synchronize their motion using hydrodynamic coupling. Inspired by the advancements in manipulating bacterial motility via physical boundaries,^[^ ^12^, ^16^, ^17^ ^]^ we build circular microcavities that trap single *E. coli* cells from a bulk population. We show that these bacteria perform a continuous circular motion, yielding periodic oscillations over hundreds of cycles that can be adjusted by engineering the cavity dimensions. When mutually coupling the microcavities with an interconnecting microchannel, we observe that the *E. coli* bacteria couple their swimming patterns and exhibit long periods of in‐phase oscillations. From a stochastic nonlinear dynamics model, we extract the coupling strength and optimize the channels to mediate synchronized oscillations between these bacterial oscillators. Our findings not only lay the foundation to engineer micro‐tools for inducing controlled oscillations and synchronization in bacterial active matter,^[^ ^18^ ^]^ but they also provide an understanding of the microscopic origins of self‐organization among the smallest living organisms.

## 2 Results and Discussion

**Figure 1:**
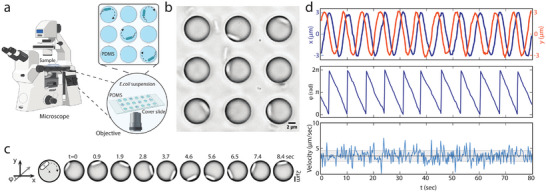
Measuring cell motility in confinement. a) Schematic of microscope setup for phase contrast imaging. b) Image of *E. coli* cells trapped in microcavities. c) Motion of a cell inside a microcavity (8µ*m* diameter). Left schematic depicts the coordinate system. Right images show a time sequence of cell positions. d) Cell coordinates and velocity. Top traces indicate x (in blue) and y (in orange) coordinates versus time; middle shows the cell phase angle; bottom show the tangential velocity, with the black line indicating the mean speed and the grey area the standard deviation.

We studied *E. coli* (delta CheA strain) bacteria swimming over an array of circular PDMS microcavities that had a diameter of *d* = 8µ*m* and a depth of 2.5µ*m* (see Experimental Section and Section SI, Supporting Information). The tendency of *E. coli* cells to move toward solid boundaries^[^ ^19^ ^]^ led to cells that were continuously swimming inside the microcavities where they performed circular paths that tracked the side walls of the cavity (See Video S1, Supporting Information). This assay provided the possibility to sieve single cells of *E. coli* from the population and study their motion in confinement. To record the motion of single cells, we used widefield phase contrast microscopy (see **Figure** **1**a,b) and tracked the position of the cells relative to the cavity (see Section SII, Supporting Information for the details). We found that *E. coli* cells that settled inside the cavities swam continuously in the clockwise direction along the cavity wall. This swimming pattern with a set chirality direction can be attributed to the right‐handedness of *E. coli* flagella which makes these bacteria “swim on the right‐hand side.”^[^ ^20^ ^]^

Figure 1c,d shows a typical trace, where a single *E. coli* cell was swimming inside an 8µ*m* microcavity with a linear speed of *v* ≈ 3.5 µ*ms* ^−1^. It can be observed that the cell performs a harmonic motion where *x* = (*d*/2)cos φ and *y* = (*d*/2)sin φ (Figure 1d) with a phase angle φ that changes uniformly in time φ = ω*t*, where the angular frequency is ω = −2*v*/*d* ≈−0.9/*s* (see also Video S2, Supporting Information). This persistent periodic behavior was observed even up to 13 min in some cases (See Figure S10, Supporting Information). Cell trapping was found to be possible for various geometries of the cavity, as cells showed clock‐wise rotations also in rectangular traps and square labyrinths (See Videos S3 and S4, Supporting Information). In rare instances, we also observed counter‐clockwise rotations of cells, which may be explained by the presence of a slip interface at the walls of the cavity.^[^ ^21^, ^22^ ^]^ The swimming pattern was also apparent in measurements conducted on cells that settled inside inverted microcavities, where *E. coli* overcame gravity and exhibited rotary motion along the ceiling (See Video S5, Supporting Information). Beyond the regular circular motion, we observed significant fluctuations in the rotary motion, see Figure 1d.

To characterize the noise, we decomposed the velocity *v* into two parts, viz., *v* = 〈*v*〉 + δ*v*, where the mean velocity 〈*v*〉 generates the periodic motion. The velocity fluctuations δ*v* from the mean velocity 〈*v*〉 were characterized as zero‐mean delta‐correlated Gaussian noise, i.e., 〈δ*v*(*t*)δ*v*(*t* + τ)〉 = 2σ^2^δ(τ), where the numerical value of σ is the standard deviation. To quantify 〈*v*〉 and σ, we performed a large number of measurements (*N* = 291) on single bacteria trapped in *d* = 8µ*m* cavities. This yielded 〈*v*〉 = 6.5 µ*ms* ^−1^ and σ = 2.6 µ*m*/*s* ^1/2^ (see Tables SII and SIII, Supporting Information for details). We note that σ^2^ in our measurements are much larger than the Brownian diffusion coefficient of ~0.1 µ*m* ^2^ *s* ^−1^,^[^ ^23^ ^]^ suggesting that the noise in our *E. coli* oscillators does not only stem from Brownian motion.

**Figure 2:**
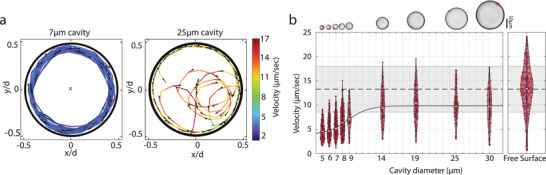
Influence of confinement size on *E. coli* motion. a) Two measured cell trajectories in a 7µ*m* (left panel) and 25µ*m* (right panel) cavity. Arrows indicate swimming direction; trajectory color indicates velocity (see scale on the right). b) Velocity distributions of *E. coli* as a function of cavity diameter. Median values are fit with a smooth curve shown with the black line. Right panel is the distribution of speed of cells swimming over a free surface. Its the mean value is indicated by the dotted line with the grey shade indicating the standard deviation.The number of measurements are *N* = 154 (*d* = 5 µ*m*), *N* = 217 (*d* = 6 µ*m*), *N* = 226 (*d* = 7 µ*m*), *N* = 291 (*d* = 8 µ*m*), *N* = 46 (*d* = 9 µ*m*), *N* = 104 (*d* = 14 µ*m*), *N* = 125 (*d* = 19 µ*m*), *N* = 100 (*d* = 25 µ*m*), *N* = 83 (*d* = 30 µ*m*), *N* = 304 (free surface).

We further found that the rotational speed of the bacteria depends on the cavity size. We performed single‐cell measurements on PDMS microcavities of diameters ranging from *d* = 5 to 30µ*m*. **Figure** **2**a shows examples of measured cell trajectories for a 7µ*m* and a 25µ*m* microcavity. Whereas cells trapped in the 7µ*m* microcavities were found to continuously follow the cavity wall similar to Figure 1c, cells in the large 25µ*m* cavities exhibited two distinct types of dynamics (see Video S6 and Figure S11, Supporting Information): trapped cells were observed to have periods of clockwise spiraling within the cavity interior, i.e. without running along the cavity walls, which alternated with periods of swimming clockwise along the cavity wall. We observed (see Figure S12, Supporting Information) that the bacterial activity in 7µ*m* cavities was almost entirely concentrated at the cavity edge (99%), while this was reduced to 77% in the 25µ*m* microcavities.

For the 7µ*m* microcavities, we found that *v* = 5.6 ± 2.2 µ*ms* ^−1^ (mean ± sd), while we observed an almost twice higher speed of *v* = 10 ± 3.3 µ*ms* ^−1^ (mean ± sd) for the 25 µ*m* microcavities. Given this sizeable difference, we quantified the speed of single‐cell rotary motion for a range of confinement sizes (Figure 2c). We found that cells slowed down significantly when driven in more strongly confined cavities, reducing their average speed from ~10 µ*ms* ^−1^ (which is 75% of the measured speed of a cell on a free surface) in cavities with diameter *d* ⩾ 14 µ*m*, to 5 µ*ms* ^−1^ for *d* ⩽ 6 µ*m*. We speculate that this drop‐off in speed occurs as the cavities become smaller than the size of an *E. coli* cell with its flagella.^[^ ^24^ ^]^ Interestingly, the cell residence time, i.e., the time cells remain trapped inside the circular well, was ≈1 *min* on average, independent of diameter (See Figure S13, Supporting Information).

**Figure 3:**
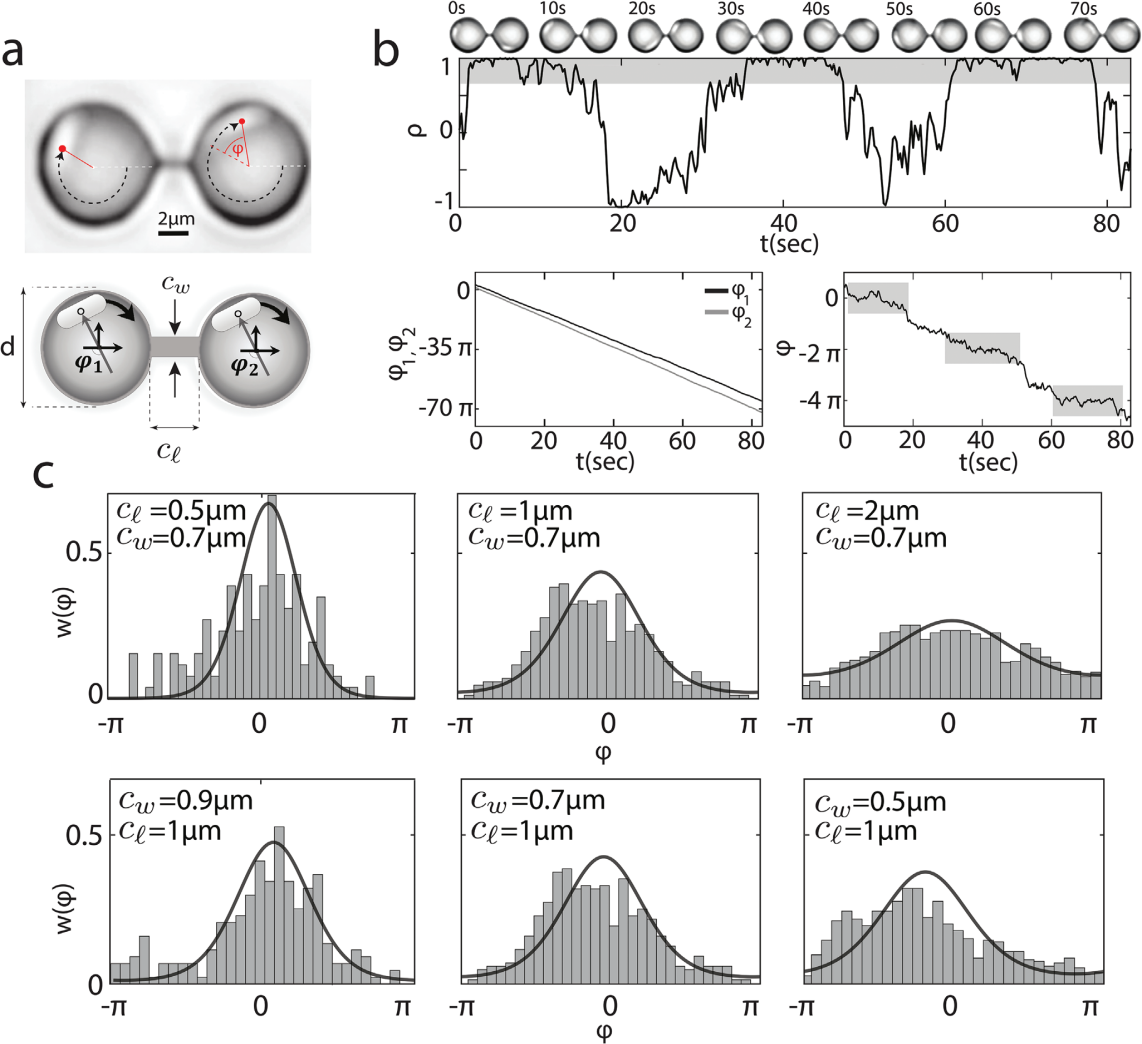
Coupled *E. coli* oscillators. a) Two identical circular microcavities of diameter *d* are connected with a channel of length *c*_ℓ_ = 0.5,1,2 µ*m* and width *c*_*w*_ = 0.5,0.7,0.9 µ*m*. b) Strong coupling is observed for the bacterial motions for *c*_*w*_ = 0.5µm, *c*_ℓ_ = 0.5µm, where ρ oscillates non‐periodically with 〈ρ〉 ≠ 0. The phase difference φ shows slow‐fast dynamics analogous to the motion of an overdamped particle with an alternating slow‐fast speed on a washboard potential. Here, grey shaded windows show the phase‐locked regions. c) Probability distribution of the phase difference φ. Solid curves are fits of von Mises distribution^[^ ^45^ ^]^ (wrapped normal distribution). In the top panels, $c_{w}=0.7\;\mu m$ and the number of data points (*n*) is *n* = 1071 $\left(c_{\ell }=0.5\;\mu m\right)$, *n* = 938 $\left(c_{\ell }=1\;\mu m\right)$, and *n* = 823 $\left(c_{\ell }=2\;\mu m\right)$. In the bottom panels, $c_{\ell }=1\;\mu m$ and *n* = 1224 $\left(c_{w}=0.9\;\mu m\right)$, *n* = 938 $\left(c_{w}=0.7\;\mu m\right)$, and *n* = 972 $\left(c_{w}=0.5\;\mu m\right)$.

Next, we investigated the motion of a *pair* of *E. coli* cells in two neighboring microcavities. We noticed that generally two cells in adjacent wells of the same size did not show signs of coupled dynamics (see Video S7, Supporting Information). The phase difference between them ran freely as a function of time ‐ despite a mutual distance that was only equal to the cavity diameter (8 µ*m*). This indicates that hydrodynamic couplings through the bulk fluid beyond the cavities were insufficient to induce synchronization. However, when we connected the cavities with a microchannel (see **Figure** **3**a), we strikingly observed that two bacteria move in unison (see Figure 3b; Video S8, Supporting Information). The channel allowed the fluid exchange between the microcavities but was too narrow to allow bacteria swim through.^[^ ^25^ ^]^

To experimentally detect the correlated motion of the bacteria, we defined a time‐dependent correlation function ρ ≡ cos φ^[^ ^26^ ^]^ in which φ = φ_2_ − φ_1_ is the measured phase difference between the two neighboring bacteria as a function of time. Figure 3b shows the result of one such experiment where two bacterial cells moved synchronously in connected microcavities (*d* = 8 µ*m*, *c*_*w*_ = 0.5µm, *c*_ℓ_ = 0.5µm). From Figure 3b, it can be observed that the motion of cells is partially phase‐locked, and the coupled dynamics involves distinct transitions from calmer epochs where ρ ≈ 1, signaling perfect synchronization, to periods of large modulations where ρ ≠ 1. This intriguing observation is reminiscent of the slow‐fast dynamics observed theoretically at the onset of synchronization.^[^ ^27^ ^]^ To explore the possibility of modulating the coupling strength, we designed channels of different length (*c*_ℓ_) and width (*c*_*w*_) and generated histograms of the phase difference (modulo 2π) between neighboring cells for a large number of connected microcavities with *d* = 7 µ*m* (see Figure 3c). We observed that the histograms became sharper for channels that were shorter, indicating a greater likelihood of achieving synchronization.

To quantify the strength of the coupled motion, we next fitted our data with the Adler equation $\dot{\varphi }=\Delta \omega -k\sin\varphi +\xi (t)$.^[^ ^28^ ^]^ Here, Δω represents the frequency mismatch Δω = ω_2_ − ω_1_ between two adjacent cells, *k* is the coupling parameter, and ξ(*t*) denotes zero‐mean delta‐correlated Gaussian noise, i.e., <ξ(*t*) > = 0 and <ξ(*t*)ξ(*t* + τ) > = 4(σ/*d*)^2^δ(τ), where σ is measured from single‐cell velocity fluctuations δ*v* and the factor 4 comes from the assumption that noise in the system is uncorrelated (see Section SIII, Supporting Information). The Adler equation, which describes the motion of an overdamped particle sliding on a washboard potential,^[^ ^29^ ^]^ is one of the simplest models for studying synchronization that can be derived from heuristic arguments (see Section SIII, Supporting Information).^[^ ^30^ ^]^ For the coupled motion of bacteria this equation can be also derived from analysis of fluid‐rotor interaction in Stokes flow, where the hydrodynamic coupling is mediated by the normal viscous force transmitted through the channel (See Section SIV, Supporting Information).

**Figure 4:**
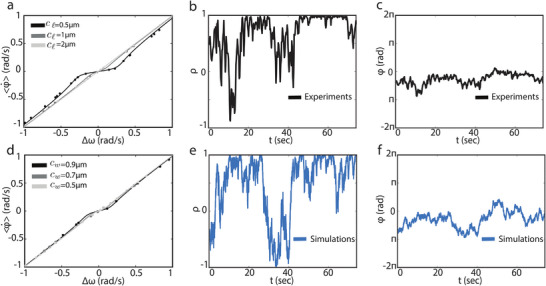
Synchronization region in connected microcavities. The synchronization range for fixed channel width $c_{w}=0.7\;\mu m$ but different channel lengths in a) and for varying channel width *c*_*w*_ but fixed channel length $c_{\ell }=1\;\mu m$ in d). Solid curves in (a) and (d) are the theoretical predictions, and the dots are experimental measurements with model‐based estimation for the frequency mismatch Δω. The number of measurements (*N*) in each curve is: *N* = 26 $\left(c_{\ell }=0.5\;\mu m\right)$, *N* = 22 $\left(c_{\ell }=1\;\mu m\right)$, *N* = 15 $\left(c_{\ell }=2\;\mu \right)$, *N* = 25 $\left(c_{w}=0.9\;\mu m\right)$, *N* = 22 $\left(c_{w}=0.7\;\mu m\right)$, and *N* = 8 $\left(c_{w}=0.5\;\mu m\right)$. b,c) Synchronized motion of coupled *E. coli* (*c*_*w*_ = 0.5µm, *c*_*l*_ = 0.5µm), where ρ = 〈ρ〉 ≈ 1, and hence, the phase difference φ is fully locked with the exception of noise‐induced fluctuations. e,f) numerical simulations of the noisy Adler integrated by the Euler–Maruyama method^[^ ^46^ ^]^ for the experimental conditions of (b) and (c), and extracted parameters *k* = 0.26 rad s^−1^, σ = 2.2 µ*m*/*s* ^1/2^, and Δω = −0.0025 rad s^−1^.

In order to obtain the coupling parameter *k* from our data, we linearized the Adler equation around the stable phase difference, which is approximately φ ≈ 0 (**Figure** **4**c), and calculated the variance 〈φ^2^〉 = (2/*k*)(σ/*d*)^2^, see Methods for details. Since we know σ for the microcavities, and experimentally measured 〈φ^2^〉, we could obtain the coupling parameter *k* for different channel dimensions (see Experimental Section for details). As expected, we found that shorter channels yielded an increased *k* while the coupling got lost with increasing *c*_ℓ_ such that for *c*_ℓ_ = 2µ*m*, only mild coupling could be observed. We also noticed that the dependence of *k* on *c*_*w*_ was minor since changing it from 0.5 to 0.9µm did not yield a noticeable increase in the coupling strength (see **Table** **1**). These findings are consistent with a minimalistic model based on Stokes flow which highlights the importance of channel for the bacteria to sync and suggests that the hydrodynamic coupling $k\propto c_{w}/c_{\ell }^{2}$ (see Experimental Section and Section SIV, Supporting Information). Experimental verification of the dependence of coupling on *c*_*w*_ was difficult as increasing the channel width beyond 0.9µm caused the bacteria to swim through.

**Table 1:** Model‐based estimation for the coupling parameter as a function of the channel width and length.

k(rad/s)	$c_{\ell }=0.5\;\mu m$	$c_{\ell }=1\;\mu m$	$c_{\ell }=2\;\mu m$
$c_{w}=0.5\;\mu m$	0.26	0.17	0.13
$c_{w}=0.7\;\mu m$	0.38	0.18	0.13
$c_{w}=0.9\;\mu m$	0.31	0.19	0.14

Using the Adler equation and the experimentally measured average rate of the phase difference $\langle \dot{\varphi }\rangle$, we also estimated the frequency mismatch Δω of the pair of *E. coli* cells moving in connected cavities (see Experimental Section). Figure 4a,d shows Shapiro‐like plateaus^[^ ^30^ ^]^ obtained by fitting the Adler equation to our data. As expected, we observed wider plateaus for shorter channels. In Figure 4b,c we report a typical correlation function ρ and phase difference φ when *k* > Δω and thus within the synchronization plateau. Video S9 (Supporting Information) shows the same synchronized *E. coli* oscillators. It can be observed that the phase difference remains in the vicinity of zero with small‐amplitude fluctuations that are induced by noise. In Figure 4e,f we also show results from the numerical integration of the noisy Adler for the same experimental configuration in which we used the estimated parameter of the experiments (σ, *k*, and Δω). These results demonstrate that single *E. coli* oscillators display emergent nonlinear dynamic behaviors in confinement through hydrodynamic forces. Interestingly, the synchronized motion we observed were also well reproduced by the simplest mathematical model of synchronization i.e, the Adler equation.

## 3 Conclusion

Summing up, we presented a platform comprising arrays of circular microcavities to study the motion of coupled *E. coli* cells in confinement. The bacteria were found to exhibit clock‐wise rotations along the cavity walls over minute time scales that could be potentially sustained indefinitely by engineering cavity dimensions. By devising sets of microcavities that were pairwise connected by channels, we showed that *E. coli* cells can coordinate their motion to neighboring cells, thus exhibiting coupled oscillatory behavior. The channels could be engineered to induce strong enough hydrodynamic coupling that led to rich nonlinear dynamic phenomena, including slow‐fast dynamics and synchronous oscillations in the presence of noise. The microcavities could be even designed to concentrate and study the synchronous motion of multiple cells in a circular cavity, analogous to runners on a race track (see Video S10, Supporting Information). Previous experimental studies already hinted at weak synchronization of *E. coli* cells from unknown origins in dense populations.^[^ ^15^ ^]^ Highly concentrated suspensions of *Bacillus subtilis* and *Pseudomonas aeruginosa* also showed emergent nonlinear dynamic behaviors including propagating spiral waves,^[^ ^31^ ^]^ active turbulence^[^ ^32^ ^]^ in bulk, and self‐organization in confinement.^[^ ^16^, ^33^ ^]^ Our single‐cell data expand on these intriguing observations, elucidating the role of hydrodynamic forces in the generation of coupling between adjacent micro‐swimmers. Furthermore, it shows that spontaneous order can be engineered and controlled at the level of single cells.

Synchronization is important in biology, physics, and engineering across different time and length scales, from planetary resonances in the solar system,^[^ ^27^ ^]^ to the synchronous flashing of fireflies,^[^ ^34^ ^]^ or even the spontaneous clapping of the audience in a theater.^[^ ^35^ ^]^ Here, we report discovery of synchronization between single bacteria, taming their random motion and engineering order out of their chaotic dynamics. These experiments provide supporting evidence for biological oscillators which obey simple models of phase synchronization. Thus, providing the basis for testing a great deal of classical works on Kuramoto model oscillators conducted thus far mainly with chemical oscillators^[^ ^36^ ^]^ toward networks of biological systems. Moreover, our results support theoretical works on hydrodynamically coupled active components,^[^ ^37^ ^]^ and call for further studies to understand, control, and unravel the stochastic nonlinear phenomena generated by micro‐swimmers in confinements. By shape optimization of the microcavities and channels, to enhance the coupling strength and suppress noise, we envision that bacterial oscillators can evolve into large arrays of synchronous microbial matter with adjustable couplings, paving the way to controllable micro‐organism‐based oscillator networks and confined active matter.^[^ ^38^ ^]^

## 4 Experimental Section

### Sample Preparation—Cell culture

Smooth sailing *Escherichia coli* (delta CheA strain) were grown in Lysogeny broth (LB). A monoclonal colony was diluted in 5mL LB and left to grow overnight at 30°*C* while gently stirred in an incubator. On the day of the experiments, an 50µ*L* aliquot was diluted 1:100 in volume into 5mL LB and grown for 2.5 h at 30°*C* to mid‐exponential phase. A subsample of this culture was again diluted typically 1:10 in fresh LB to finally reach an optical density (OD600) of 0.05.

### Sample Preparation—Mold preparation

Molds were prepared from 500 micron thick silicon wafers with a 285nm thick layer of silicon dioxide. The wafers were patterned by electron beam lithography followed by reactive ion etching, resulting in a feature height of 2.5 micron for PDMS casting.

### Sample Preparation—PDMS Preparation

Pre‐polymer PDMS was mixed with curing agent in 4:1 ratio (2g PDMS to 0.5g curing agent), and stirred manually with the tip of a sterile pipette. The mixture was then degassed in a vacuum chamber for 10–15 min, until all air bubbles had disappeared. Using the tip of a pipette, a small amount of PDMS was carefully dipped in the center of one of the structures of the patterned wafer (see Section SI, Supporting Information). Then, a 1mm thick, 22 × 22mm glass slide was placed on the droplet, spreading it over the patterned wafer structure. The assembly was subsequently baked at 90°*C* for 2.5h in an oven. Prior to baking, the silicon wafer was pre‐treated with (Tridecafluoro‐1,1,2,2‐tetrahydrooctyl)trichlorosilane (FTS) to ensure that the PDMS mould could be easily released after it had been cured.^[^ ^39^ ^]^ With a sharp sterile razor blade, the glass slide was carefully lifted from the wafer and stored till the day of the experiment. The PDMS stuctures were eventually plasma treated at 20W power and 60mTorr oxygen chamber pressure for 30s in order to make them hydrophyllic.

### Sample Preparation—Imaging

Measurements were carried out using an inverted microscope (Nikon Ti) under bright‐field illumination, through a 100× oil‐immersion objective. Recordings typically had 2min duration at frame acquisition interval of 0.23s. For imaging, ≈35 µ*L* cell culture was pipetted onto a prepared PDMS sample and placed on a coverslip holder. Then, with parafilm a sample cover was placed over the PDMS sample, creating a sealed chamber (see Figure 1a). The assembly was placed inside a closed microscopic chamber maintained at 30°*C*.

### Model‐Based Estimation—Estimation of the Coupling Parameter in Connected Microcavities

To estimate the coupling parameter, the Adler equation was linearized around φ = 0, and obtain

(1) $$\begin{array}{c} \dot{\varphi }=-k\varphi +\xi (t) \end{array}$$

Equation (1) can be readily integrated to yield

(2) $$\begin{array}{cc} \varphi (t) & =\varphi \left(0\right)e^{-kt}+\int_{0}^{t}\xi \left(\tau \right)e^{k(\tau -t)}d\tau \end{array}$$

with a rapidly vanishing mean value 〈φ〉 = φ(0)*e* ^−*kt*^. Thus, at steady‐state, where 〈φ〉 = 0, the variance is given by

(3) $$\begin{array}{cc} \left\langle \varphi^{2}\right\rangle & =\int_{0}^{t}\int_{0}^{t}e^{k(\tau -t)}e^{k(s-t)}\left\langle \xi \left(\tau \right)\xi \left(s\right)\right\rangle d\tau ds=4{\left(\frac{\sigma }{d}\right)}^{2}\int_{0}^{t}e^{2k(\tau -t)}d\tau \end{array}$$

which yields the steady‐state variance 〈φ^2^〉 = 2σ^2^/(*kd* ^2^), and can be used to estimate the coupling parameter *k* = 2σ^2^/(〈φ^2^〉*d* ^2^). Using the measured noise intensity 2(σ/*d*)^2^ and the variance of the locked phase difference, the following estimations were obtained for the coupling parameter

### Model‐Based Estimation—Estimation of the Frequency Mismatch between Coupled *E. coli* Cells

To estimate the frequency mismatch of the pair of *E. coli* cells, the probability density of the phase difference *w*(φ, *t*) of the Adler equation was analyzed, which obeys the following Fokker–Planck equation^[^ ^40^ ^]^

(4) $$\frac{\partial w}{\partial t}=-\frac{\partial }{\partial \varphi }\left[(\Delta \omega -k\sin\varphi )w\right]+4{\left(\frac{\sigma }{d}\right)}^{2}\frac{\partial^{2}w}{\partial \varphi^{2}}$$

and expresses the conservation of probability ∂*w*/∂*t* + ∂*J*/∂φ = 0, where

(5) $$J=\left(\Delta \omega -k\sin\varphi \right)w-4{\left(\frac{\sigma }{d}\right)}^{2}\frac{\partial w}{\partial \varphi }$$

is the probability current. A stationary (time‐independent) probability density *w*_ss_ was sought, where ∂*w*_ss_/∂*t* = 0. The stationary solution of Equation (4) is 2π‐periodic in φ, and therefore, it can be written in the terms of its Fourier expansion^[^ ^30^ ^]^ $w_{\mathrm{ss}}=\sum_{-\infty }^{\infty }W_{n}e^{in\varphi }$. From the normalization condition $\int_{0}^{2\pi }w_{\mathrm{ss}}\left(\varphi \right)d\varphi =1$, it was found that *W*_0_ = (2π)^−1^, and from the reality of *w*_ss_, it was deduced that $W_{-n}=W_{n}^{*}$, where $W_{n}^{*}$ is the complex‐conjugate of *W*_*n*_. Furthermore, by integrating Equation (5) with respect to φ from 0 to 2π, it was found that

(6) $$\begin{array}{c} 2\pi J=\int_{0}^{2\pi }\left(\Delta \omega -k\sin\varphi \right)w_{\mathrm{ss}}\left(\varphi \right)d\varphi -4{\left(\frac{\sigma }{d}\right)}^{2}\left[w_{\mathrm{ss}}\left(2\pi \right)-w_{\mathrm{ss}}\left(0\right)\right]\\ =\int_{0}^{2\pi }\left(\Delta \omega -k\sin\varphi \right)w_{\mathrm{ss}}\left(\varphi \right)d\varphi \equiv \left\langle \Delta \omega -k\sin\varphi \right\rangle =\left\langle \dot{\varphi }\right\rangle \end{array}$$

Substitution of the Fourier expansion into Equation (5), yields the following tridiagonal system of equations 

(7) $$-\left(\frac{4in\sigma^{2}}{d^{2}}-\Delta \omega \right)W_{n}-\frac{ik}{2}\left(W_{n-1}-W_{n+1}\right)=J\delta_{n,0}$$

From Equation (7), the following relation for *n* > 0 was found,

(8) $$\frac{W_{n}}{W_{n-1}}={\left[-\left(\frac{8n\sigma }{kd}+\frac{2i\Delta \omega }{k}\right)+\frac{W_{n+1}}{W_{n}}\right]}^{-1}$$

Setting *n* = 1, and using continuous fractions, the following rapidly converging solution^[^ ^41^ ^]^ was obtained

(9) $$W_{1}=\frac{{\left(2\pi \right)}^{-1}}{-\left(\frac{8\sigma^{2}}{kd^{2}}+\frac{2i\Delta \omega }{k}\right)+\frac{1}{-\left(\frac{16\sigma^{2}}{kd^{2}}+\frac{2i\Delta \omega }{k}\right)+\frac{1}{-\left(\frac{24\sigma^{2}}{kd^{2}}+\frac{2i\Delta \omega }{k}\right)+\cdots }}}$$

By setting *n* = 0 in Equation (7), it was found that *J* = Δω/(2π) − *k*ℑ{*W*_1_}, and therefore, using Equation (6), It is obtained

(10) $$\begin{array}{c} \langle \dot{\varphi }\rangle =\Delta \omega -2\pi kI\{W_{1}\}=\Delta \omega \\ \;+\;kI\left\{{\left[\left(\frac{8\sigma^{2}}{kd^{2}}+\frac{2i\Delta \omega }{k}\right)+\frac{1}{\left(\frac{16\sigma^{2}}{kd^{2}}+\frac{2i\Delta \omega }{k}\right)+\frac{1}{\left(\frac{24\sigma^{2}}{kd^{2}}+\frac{2i\Delta \omega }{k}\right)+\cdots }}\right]}^{-1}\right\} \end{array}$$

Since the only unknown quantity in Equation (10) is Δω (σ is estimated from a single‐cell, and *k* is estimated independently from the variance as described above), Equation (10) can be used to estimate Δω with increasing accuracy as the number of iterations in the continuous fraction was increased.

### Minimalistic Model for Hydrodynamic Synchronization

To understand the role of hydrodynamics in the synchronization of *E. coli* cells, we model them as force dipoles.^[^ ^42^ ^]^ The thrust force of each cell is defined as **F** = *F* **e**, where *F* is the magnitude, which is assumed to be constant, and **e** is the direction of the force (see Section SIV, Supporting Information). For a pair of cells in adjacent cavities connected by a microchannel, a local normal vector **n**_*i*_ = (cos φ_*i*_, sin φ_*i*_) and tangential vector **t**_*i*_ = (sin φ_*i*_, −cos φ_*i*_) were defined where *i* = 1, 2 to track the position of each cell (see Figure 3a). The measured velocity of each cell can be written as $v_{i}=\left(d/2\right)\dot{\varphi_{i}}t_{i}$ assuming that the cells move only on a circular trajectory. This velocity is the sum of self interaction of the cell, given by (*d*/2)ω_*i*_ **t**_*i*_ and interaction due to the other cell, given by **G · F** where **G** is the Oseen tensor and force **F** of the neighbouring cell (see Section SIV, Supporting Information). The velocity of each cell can thus be written as,

(11) $$\begin{array}{cc} \frac{d}{2}\dot{\varphi_{1}} & =\frac{d}{2}\omega_{1}+t_{1}\cdot G\cdot F_{2} \end{array}$$

(12) $$\begin{array}{cc} \frac{d}{2}\dot{\varphi_{2}} & =\frac{d}{2}\omega_{2}+t_{2}\cdot G\cdot F_{1} \end{array}$$

The experiments suggest that the mechanism of interaction between the two cells is primarily mediated by the channel. Therefore, for simplicity, the channel was considered as two flat plates with length *c*_ℓ_ distant apart by width *c*_*w*_. The flow field between the flat plates can be approximated as a 2D source dipole,^[^ ^43^ ^]^ with a strength $c_{w}^{2}F/2$ ^[^ ^44^ ^]^ (see Section SIV, Supporting Information for details). Subtracting Equations (12) and (11) leads to

(13) $$\dot{\varphi }=\Delta \omega -\frac{3c_{w}F}{16\pi \mu d(d+c_{\ell })2}\sin\varphi$$

where φ = φ_2_ − φ_1_ and μ is the viscosity of the fluid. It was noted that Equation (13) has the same form of the Adler equation, with the coupling term *k* expressed in terms of physical parameters of the system. It can be seen that the coupling parameter *k* is proportional to the channel width but inversely proportional to the square of the distance between the two cells.

## Conflict of Interest

Yes; Employment or leadership: A.J., I.E.R; SoundCell B.V. Consultant or advisory role: F.A.; SoundCell B.V. The authors declare no further competing interests.

## Author Contributions

A.J., and F.A. conceived the idea. A.J. and V.S. collected the data and performed the experiments. I.E.R fabricated the silicon traps. A.J, and V.S. performed the bacterial manipulation. O.S. formulated the theoretical modeling, conducted analytical and numerical analysis, and performed the fitting with the experimental data. K.S. developed the hydrodynamic model. A.J. F.A. and V.S. designed the experiments. The project was supervised by F.A, C.D. All authors contributed to the data analysis, interpretation of the results, writing of the manuscript, with the main contribution from FA.

## Supporting information

Supporting Information

Supplemental Video 1

Supplemental Video 2

Supplemental Video 3

Supplemental Video 4

Supplemental Video 5

Supplemental Video 6

Supplemental Video 7

Supplemental Video 8

Supplemental Video 9

Supplemental Video 10

## Data Availability

The data that support the findings of this study are available from the corresponding author upon reasonable request.
