# Supplementary material for: Synchronization of E. coli Bacteria Moving in Coupled Microwells
Source: Small. 2024 Nov 25;21(3):2407832. doi: 10.1002/smll.202407832 (PMC11753501; doi:10.1002/smll.202407832)
Supplement: Supplementary file 1 — Supporting Information [file SMLL-21-2407832-s002.pdf]

NANO · MICRO
small

Supporting Information

for *Small*, DOI 10.1002/smll.202407832

Synchronization of *E. coli* Bacteria Moving in Coupled Microwells

*Aleksandre Japaridze, Victor Struijk, Kushal Swamy, Ireneusz Rosłoń, Oriel Shoshani, Cees Dekker and Farbod Alijani**

Supplementary Information: Synchronization of *E. coli* bacteria moving in coupled microwells

A. Japaridze,[1,2] V. Struijk,[1] K. Swamy,[1] I. E. Rosłoń,[1,2] O. Shoshani,[3] C. Dekker,[1] and F. Alijani[1,4]

[1] *Delft University of Technology, Delft The Netherlands*
[2] *SoundCell B.V. Delft, The Netherlands*
[3] *Ben-Gurion University of the Negev, Beer-Sheva, Israel*
[4] *Corresponding author: f.alijani@tudelft.nl*

I. FABRICATION OF MICROCAVITIES

To obtain an array of circular microcavities, first a master was fabricated with the exact inverted shape. This was done by means of dry-etching. The master consisted of a silicon (Si) wafer, with a 285 nm surface layer of silicon dioxide (SiO2). On the wafer surface multiple arrays of micropillars were patterned, every array with a specific pillar diameter. Figure S1 shows a scanning electron microscope (SEM) image of the patterned wafer surface.

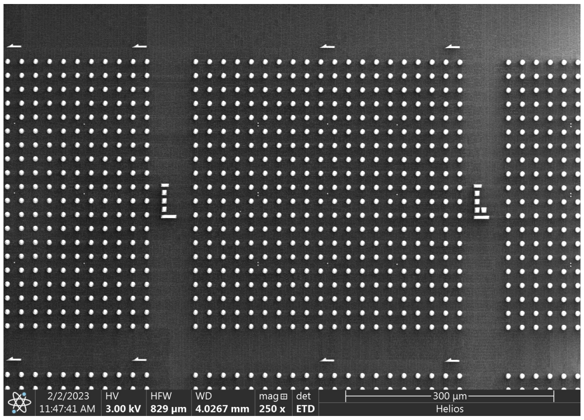

Fig S1: Scanning Electron Microscope (SEM) image showing an array of micropillars that act as the master for the fabrication of microcavities

In the process of preparing the wafer for PDMS casting, it was necessary to apply a lubricating surface coating in order to ensure that the mould would be easily released after curing. In this particular case, we used (Tridecafluoro-l,l,2,2-tetrahydrooctyl)trichlorosilane (FTS) as silanization reagent, which has previously been found to form low-friction monolayers on silicon devices [1]. To apply the FTS coating, an open container with a few droplets of FTS was placed together with the patterned wafer in a closed vacuum chamber for 15min. During this process, the FTS vaporized, and the resulting molecules formed covalent bonds with silicon, resulting in the formation of a thin lubrication film on the wafer surface. The wafer silanization was performed periodically every ∼10-15 release cycles. While the coating was initially applied as a finishing treatment in the wafer preparation process, periodic re-application ensured that it continues to act as an effective lubricant for the mould.

A. Substrate preparation

In our study we used PDMS due to its adjustable mechanical, physical and tribological properties, in particular through altering curing agent mixing ratio and the type of heat treatment used for curing [2]. This allowed us to conjure up a suitable fabrication recipe for our application. For microcavities 2.5 μm deep and 5-30 μm in diameter we found that pre-polymer PDMS : curing agent mixing ratio of 4:1, along with heat treatment of 2.5 hours at 90 ^oC (in the oven) is suitable. After curing, the substrate was lifted by carefully wedging a sharp sterile razor blade between wafer and glass slide, as shown in figure S2 (f). For this lifting technique it was important to have at least one PDMS-free corner underneath the slide to function as starting edge for the blade. For a slide of 22x22mm that means the volume of PDMS dispensed on the patterned structure should be approximately 1 μl. To lift the substrate the blade was simply moved upwards, which created the required peeling forces for the PDMS layer to slowly release

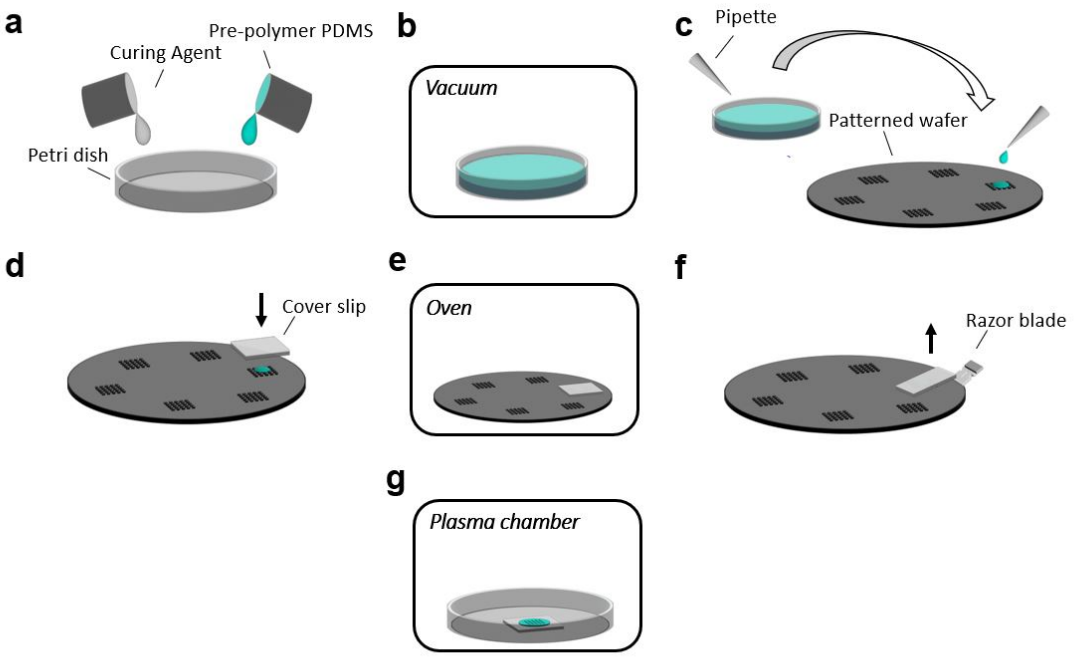

Fig S2: The process of fabricating PDMS substrates with microcavities, PDMS is colored green for visibility. (a) Liquid pre-polymer PDMS is mixed with a curing agent and thoroughly stirred. (b) The mixture is placed under vacuum until visibly all air-bubbles are removed. (c) A droplet of PDMS is dispensed on each of the patterned structures on the wafer, for explanatory reason here just one is shown. (d) By placing a glass cover slip on top, the droplet spreads out over the patterned wafer structure and ends up covering it entirely. (e) The assembly is placed inside an oven to cure the PDMS film sandwiched between cover slip and wafer. (f) With a sterile razor blade the cover slip is carefully lifted from the wafer. The PDMS layer adheres stronger to glass than the silanized wafer surface, and thus releases completely from the patterned structure. (g) the substrate is placed in a plasma chamber to be surface treated by oxygen plasma.

from the wafer. Finally, in order to allow the water based bacterial solution to fill the microcavities it was necessary to overcome the hydrophobic nature of the PDMS surface. To resolve this, we did oxygen plasma surface treatment for 30 seconds, with $20W$ plasma power and $60mTorr$ oxygen chamber pressure.

B. Single and coupled microcavities

Two separate patterned wafers were used for the fabrication of substrates for experiments on bacterial trapping. One wafer was equipped with arrays of 1.5μm protruding micropillars with diameters ranging from 5-8μm, whereas the second wafer was etched to 2.5μm depth and hosted both micropillars with 8-30μm diameter, as well as dumbbell structures. Both silicon wafers were patterned following a similar dry-etching process. Dumbbell structures in this context consisted of a symmetric pair of bridged micropillars, which were fabricated with pillars ranging from 5-10μm in diameter and bridges of 0.5-1-2μm length and 0.5-0.7-0.9μm width.

Figure S3 draws an overview of the structure designs (left panels) compared to the etched wafer patterns (middle panels) and the resulting traps on the substrate (right panels). Micropillars on both wafers were designed to be spaced 4μm apart, regardless of pillar size 'd' (see left panel of fig S3a). Under 100x magnification, translating to a microscopic field of view of $\sim$133x133μm, this meant up to 225 cavities of d =5μm could be measured simultaneously, whereas up to 16 cavities of d =30μm. Dumbbell structures were designed to be spaced a pillar diameter from one another (see left panel of fig S3b), in order to minimize potential hydrodynamic interaction between cells trapped in different pairs of connected cavities.

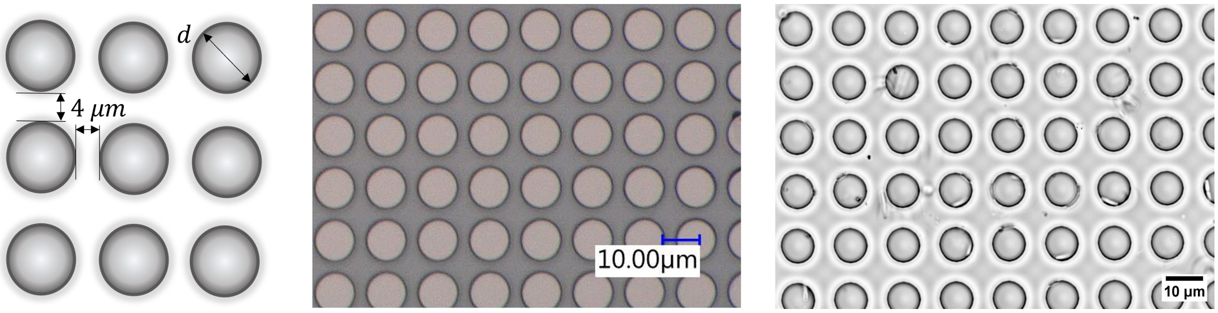

(a) Array of microcavities with $d = 10\mu m$

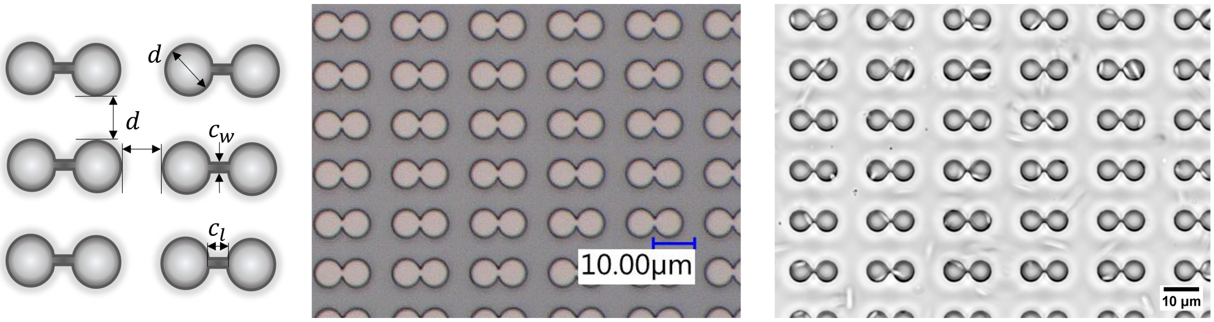

(b) Array of connected microcavities with $d = 7\mu m$, $c_w = 0.9\mu m$ and $c_w = 0.5\mu m$

Fig S3: Comparison of structure designs to the patterned wafer arrays and the resulting arrays of traps on the PDMS substrate. Panels show schematics of array dimensions (left), etched wafer patterns (middle) and corresponding traps on the PDMS substrate (right).

II. MEASURING CELL MOTION IN MICROCAVITIES

When cells were trapped in a circular microcavity they systematically moved along the wall in clockwise direction, as has been explained in the main article. In order to analyse how this rotary motion of cells was affected by the cavity size, it was necessary to have a method to track cell motion in an automated way. There exist already various cell tracking tools which are able to detect cells and track their position over time. Some of them use complex algorithms meant to segment individual cells from a cluster [3], or to track swimming cells which can suddenly turn and bump into neighbours [4]. Otherwise when there is a high degree of overlapping or a certain variability in cell phenotype, several machine learning techniques have been applied and shown to perform well [5][6]. The benefit of the way of measuring confined bacterial motion presented here however, is that it removes much of the programming complexity surrounding clustering and overlapping. Here the considered microcavities contain only a single physically isolated cell, of which the shape features are known beforehand. For this reason we chose to develop a custom tracking algorithm, which was able to track the motion of individual cells inside cavities.

The goal of the image post processing software was to track cell movement while cells were trapped inside a cavity. The first task at hand was to find all cavity locations in the microscope recording so that the positions of cells inside the cavities could be determined afterwards. Since already a single 2 minute microscope recording of cell trapping consists of approximately 600 images, finding both cavity and cell locations in each frame quickly becomes computationally intensive. Therefore, by assuming negligible camera drifts, we only measured cavity positions once for each recording. Furthermore,in image processing, instances were multiple cells were found inside the same cavity were discarded. In summary the strategy for cell tracking was as follows: First cavity positions were derived from the first frame of the recording. Then for each spotted cavity, in case there was a single cell inside, the position of the trapped cell was measured with respect to the cavity center. The latter being repeated for each frame for the duration of the recording. Figure S4 gives an overview of the cell tracking process.

Generally with object detection algorithms in image processing it is necessary to find a feature that differentiates the object in question from its surroundings. In case of the cavities in the microscopic image shown in figure S4a, it stands out that each cavity boundary was accompanied by a distinct dark ring. This area lining the inside of the cavity border had a different light reflection properties than other parts of the substrate, which offers opportunity for detection. The tracking software takes advantage of this feature by application of a pixel intensity threshold,

which filters pixels belonging to dark cavity rings from the background and converts the original image to binary (figure S4b). In the binary image the only remaining pixels were those which made up cavity rings. Next, chains of connected pixels were given a specific label which in turn created objects. Pixels with label 1 made up one object, pixels labelled 2 made up another, and so on. From each created object the location of the geometric center in the image was calculated along with the height and width, figure S4c shows the end result. These were the cavity specifics that were computed only once for every recording to be then stored in a table. As a final step we checked whether the found object dimensions lie in margin of what was expected from the known cavity diameter. Objects that did not comply were discarded, whereas objects that do can be considered for further tracking purposes. At these object locations portions were cropped from the image corresponding with single cavities. This process was then repeated for every frame in the recording, as shown in figure S4d. Ultimately for every frame of a cavity a second specialized detection algorithm looks for residing cells, and measures the position of their center of mass with respect to the cavity center. Figure S4e depicts a series of cavity frames in which a cell was found and tracked.

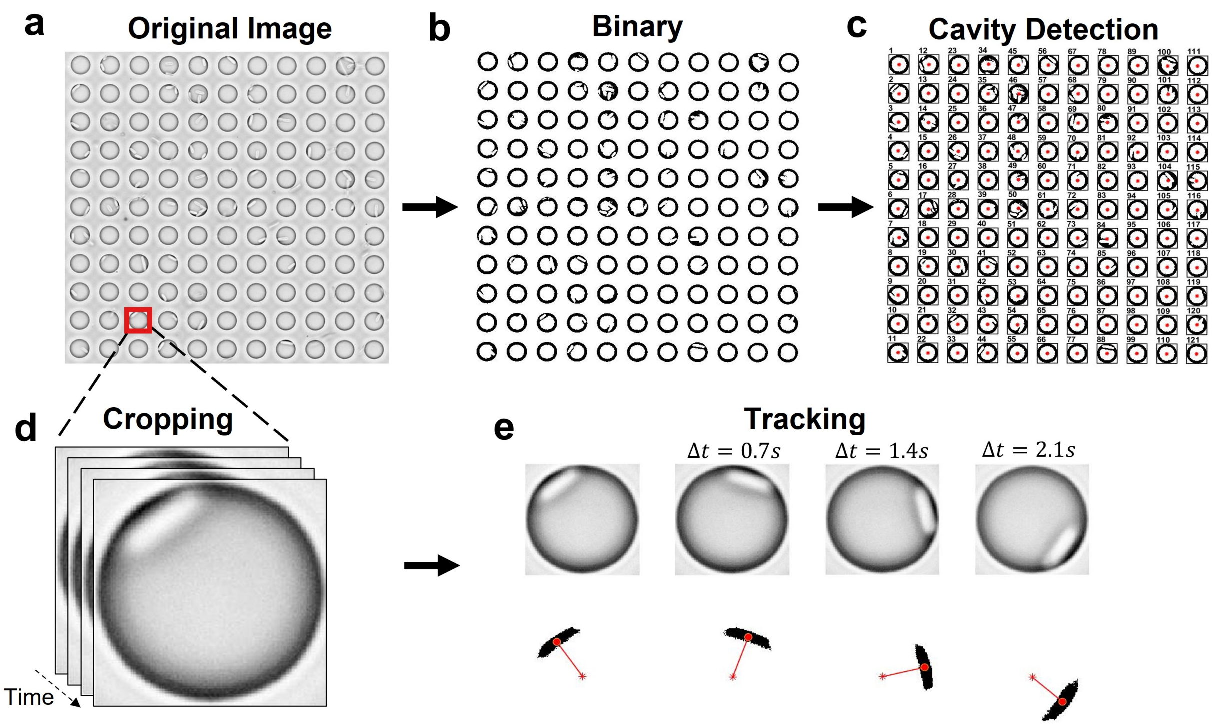

Fig S4: Flowchart of the cell tracking process. (a) A single frame taken from the microscope recording of the microcavity array. (b) Original image is converted to binary by applying a pixel intensity threshold. (c) Objects are identified as cavities from which both the geometrical center (red stars) and size can be measured. (d) When all cavities are found take cropped images of each cavity for every frame of the recording. (e) Detect cells residing inside the cavity and in case a single cell is found track its position over time

The detection of cells inside cavities was done in similar manner as the detection of cavities in the array. Figure S5 draws an overview of the process.

From a portion of the original microscopic image cropped around a single cavity (figure S5a), the goal was to detect any potential residing cells and find their position with respect to the cavity center. First, based on a circular Hough transform, a common feature extraction technique for finding circular features in imperfect images [7], the algorithm detects the edge of the cavity in consideration. In this way both the radius and geometric center were measured in a more sophisticated way than was done in the coarse cavity detection process elaborated in figure S4. Once the cavity edge was found, a mask was applied which clears the corners of the image which lie outside the cavity. In this way, cells which may have appeared in the cropped image but were not trapped were excluded from being detected. The found cavity edge and geometric center were annotated in the mask image in figure S5b as a red ring and marker respectively. Next, since *E.coli* cells appeared as the brightest feature in the image, a basic pixel intensity threshold was applied to convert the image to binary, shown in figure S5c. Connected pixels were sorted into objects, labeled with pseudo random colors in figure S5d. Making use of the known typical shape characteristics of *E.coli*, e.g. its

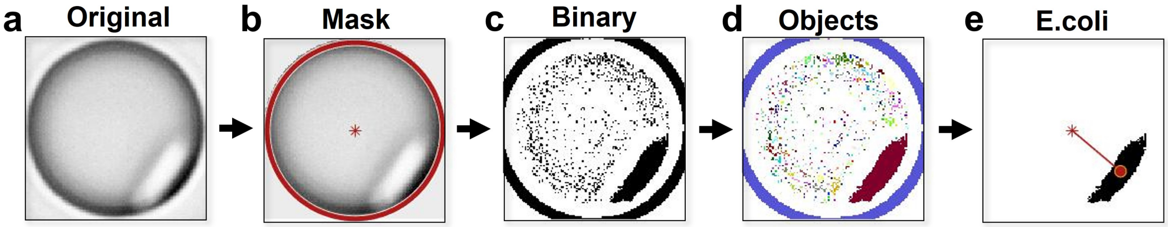

Fig S5: Flowchart of the cell detection process. (a) Image of a single cavity cropped from the array. (b) Image after application of a mask with annotated detected cavity edge (red ring) and cavity center (red star). (c) Conversion to binary through a pixel intensity threshold. (d) Groups of connected pixels labeled as objects with pseudo random colors. (e) The object representing an *E.coli* cell is filtered from the cluster and its center of mass measured relative to the cavity center

cylindrical shaped cell body and roughly 2 μm^2 size, the bacterium was finally filtered from the cluster of objects. The position of the bacterium's center of mass relative to the cavity center was then measured and stored, as shown in figure S5e, after which the next frame was loaded to repeat the process from start.

A. Cell tracking in case of multiple bacteria in cavities

	$d \leq 10$µm	$d > 10$µm
Consider	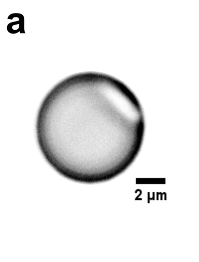	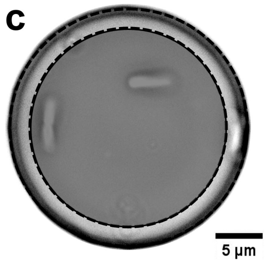
Discard	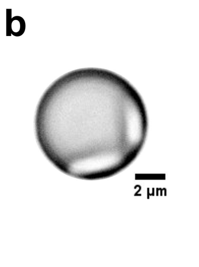	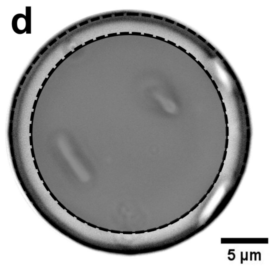

TABLE I: Overview of considered measurements versus discarded ones for cavities with diameter 'd' $\leq 10\mu m$ and $>10\mu m$. (a) A cavity ($d = 7\mu m$) with a single trapped cell. (b) A cavity ($d = 7\mu m$) with multiple trapped cells. (c) A cavity ($d = 25\mu m$) with multiple trapped cells, but only a single one located at the edge, outside of the area annotated with the dark circle. (d) A cavity ($d = 25\mu m$) with multiple trapped cells, having multiple cells located at the cavity edge

Multiple cells trapped simultaneously in the same microcavity may potentially influence each others movement. Since physical cell-cell interaction is an unwanted factor in studying the relation of cell motility to cavity size, there was no need to equip the tracking algorithm with tools to distinguish between cells. Cropped microcavity images in which more than one cell were detected were therefore omitted from further analysis. In cavities with a diameter less than the typical size of *E.coli* including flagella (approx. $10\mu m$), this measure was found to not significantly affect the quality of tracking data. Cavities of this size often hosted a single cell, and at times where a second cell jumped in, one of the two was often quickly forced out. However, with increasing diameter, cavities on average became more crowded,

and limiting the analysis to cavities with a single trapped cell rendered most measurements unusable. Thus, in order to work around the problem of crowding in large wells, only the number of detected cells that swim along the cavity wall were counted. Instances where a single cell was detected at the cavity edge were considered for further analysis, whereas measurements where multiple cells were found in this area were discarded. Table I draws an overview for the strategy used to distinguish between measurements fit for further consideration, and measurements to be discarded. For cavities $>10\mu m$, the dark circles displayed in table I are centered with the cavity and set to a diameter of 80% the cavity diameter. For the measurement to be considered, there must be exactly one cell detected with a centroid outside the circle of occlusion.

B. Data segmentation

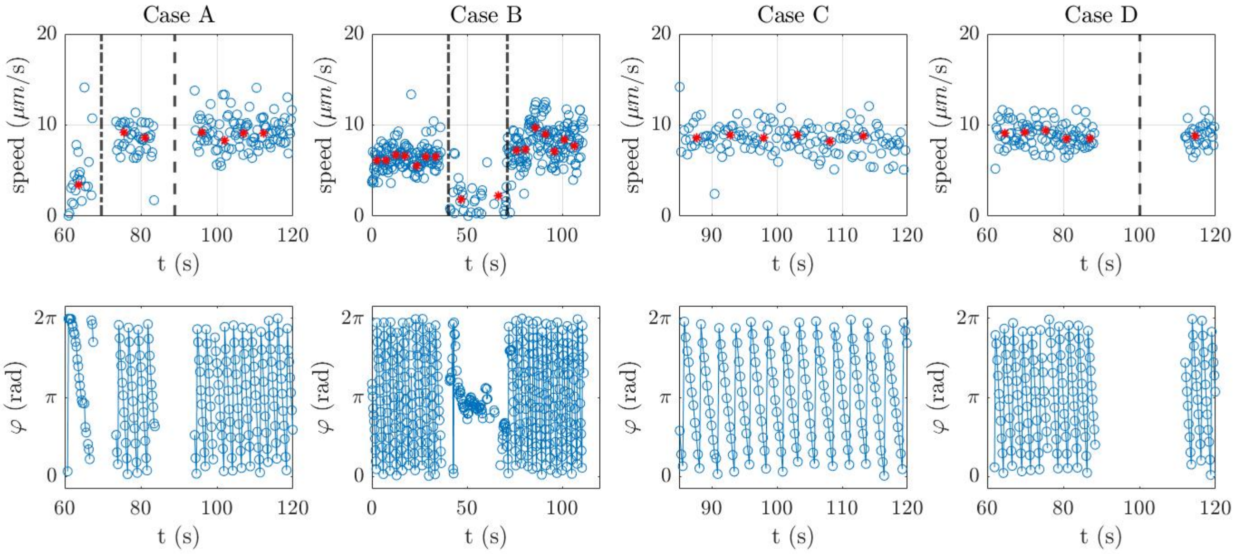

Fig S6: Four examples of how cell tracking data is analyzed and segmented, implementing the coordinate system displayed in Figure 1 of the main text. The top row diagrams show cell speed (in scatter) over time, with the corresponding cell phase angle on the bottom row. Breaks are inserted where the gap in median anchors (red markers) is more than 1.5 times the standard deviation (dashdotted black line), or where gap between velocity points was more than 10 seconds (dashed black line).

Over the duration of a typical 2 minute recording of a microcavity it is possible that the gathered tracking data consists of different cells that were trapped subsequently. In order to extract information about individual cells we therefore aimed to segment cell tracking data into individual cell runs. To achieve this, we used a segmentation method that involved computing median anchors for every 20 velocity data points. If the gap between anchors was more than 1.5 times the velocity standard deviation, or if the gap between velocity points was more than 10 seconds, a break was inserted in the data, as shown in figure S6. This ensured that the data was broken up into segments that corresponded to distinct periods of cell movement. For each data segment, we further checked whether the tracked cell was swimming by examining the phase slope. Specifically, we checked that the phase slope was negative at least more than 75 percent of the time. This check was important to ensure that we were only analyzing data from periods when the cell was actively swimming along the cavity wall, as opposed to when it was stationary or twitching due to becoming stuck.

III. THEORY AND MODEL-BASED ESTIMATION

The Adler model is a fundamental mathematical model that has been widely used to study synchronization in coupled oscillatory systems. Originally proposed by Robert Adler in 1946 to study entrainment[8], this equation has been applied to a wide range of phenomena, from electrical circuits [9] to neuronal networks [10] and the pattern formation in populations of bacteria [11]. Its ability to extract coupling strength from observed behaviors makes it a

valuable tool for understanding the mechanisms that underlie synchronization in a wide range of contexts. Here it is used to model the coupled behaviour between the cyclic motion of single cells.

Two *E.coli* trapped at opposing sides of a connected pair of cavities can be described as a set of $N = 2$ rotors, running around circular tracks, being fully specified in position by the phase angle their center of mass makes with the right side horizontal. Let ϕ_1 and ϕ_2 be the phases of the two cells, and assume that they rotate in a clockwise direction with their respective frequencies ω_1 and ω_2, as shown in Figure S7. Then the phase difference $\phi = \phi_2 - \phi_1$ between the two cells evolves according to a first-order differential equation of the form $\dot{\phi} = f(\phi)$. Since $f(\phi)$ must be 2π-periodic, i.e., $f(\phi) = f(\phi + 2\pi)$, we can express it in terms of its Fourier expansion $f(\phi) = c_0 - \sum_{n=1}^{\infty} c_n \sin(n\phi - \theta_n)$. For a leading order approximation, we truncate the Fourier expansion at the fundamental harmonic ($n = 1$), $f(\phi) \approx c_0 - c_1 \sin(\phi - \theta_1)$, define $\varphi \equiv \phi - \theta_1$, $\Delta\omega \equiv c_0$, $k \equiv c_1$, and obtain the well-known Adler equation

$$\dot{\varphi} = \Delta\omega - k \sin\varphi.$$

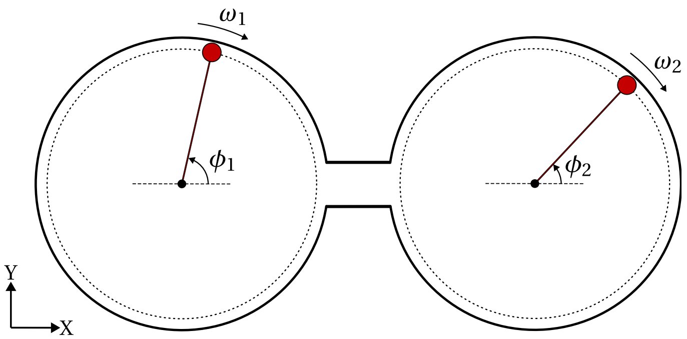

Fig S7: Schematic model of a pair of cells in connected cavities, described by particles running around circular tracks. The spherical particles represent each cell's center of mass, with ω_1 and ω_2 their respective natural frequencies and the phase angles φ_1 and φ_2, which are measured with respect to the right horizontal.

To introduce noise to the Adler equation, we consider the phase dynamics of a pair of noisy coupled *E.coli* rotors,

$$\dot{\phi}_1 = \omega_1 - k \sin(\phi_1 - \phi_2 - \theta_1) - \frac{2\delta v}{d}, \tag{1}$$

$$\dot{\phi}_2 = \omega_2 - k \sin(\phi_2 - \phi_1 - \theta_1) - \frac{2\delta v}{d}, \tag{2}$$

where δv is the velocity fluctuations term of a single bacterium oscillator as described in the main text with an intensity of $\langle \delta v(t) \delta v(t+\tau) \rangle = 2\sigma^2 \delta(\tau)$, with σ^2 being a diffusion coefficient and the standard deviation values given in Table II below. To relate the standard deviation δv to σ, we use the Wiener-Kinchin theorem which states that the spectral density of a random process is the Fourier transform of the autocorrelation with a zero frequency. The variance is then $\langle \delta v \rangle^2 = (1/2) \int_{-\infty}^{\infty} 2\sigma^2 \delta(\tau) d\tau = \sigma^2$. Therefore, the variance and σ^2 have the same numerical value but different units. Now, by subtracting Eq. (1) from Eq. (2), we obtain the noisy Adler equation of the main text $\dot{\varphi} = \Delta\omega - k \sin\varphi + \xi(t)$.

Well diameter	#Measurements	Mean ± std ($\mu m/s$)
$5\mu m$	154	4.7 ± 1.7
$6\mu m$	217	5.1 ± 2.1
$7\mu m$	226	5.6 ± 2.2
$8\mu m$	291	6.5 ± 2.6
$9\mu m$	46	7.6 ± 2.6
$14\mu m$	104	9.7 ± 3.3
$19\mu m$	125	10 ± 3.5
$25\mu m$	100	10 ± 3.3
$30\mu m$	83	9.5 ± 3.7
Surface	304	13 ± 4.7

TABLE II: Measured cell velocities in different-sized confinements compared to that on a surface. The table presents accompanying data to Figure 2b in the main text. The standard deviation contains the same numerical value of σ.

As described in the main text, the uncoupled motion is associated with $\langle\rho\rangle \approx 0$, and coupled in-phase synchronized motion is associated with $\langle\rho\rangle \approx 1$, where $\langle\rho\rangle$ denotes the expected value of ρ. We conducted numerical simulations of the noisy Adler equation to find the threshold value of $\langle\rho\rangle$ in which the system dynamics transition from uncoupled to coupled motion. We found that $\langle\rho\rangle \approx 0.5$ can be considered a good marker for the transition to slow-fast dynamics (see Figure S8) that stem from the critical slowing down near bifurcation phenomenon [12]. Therefore, using numerical simulations of the noisy Adler equation, we numerically identified the saddle-node bifurcation in which the transition to synchronization occurs in the deterministic Adler equation.

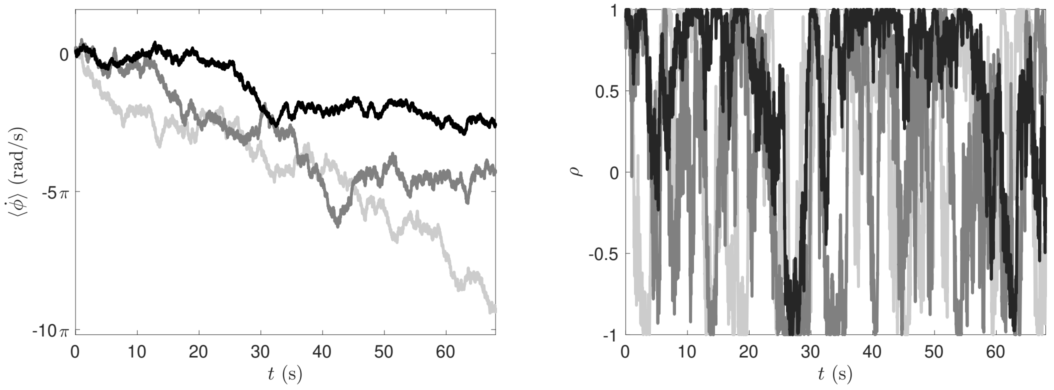

Fig S8: Transition from uncoupled to coupled motion. From numerical simulations of the noisy Adler equation [with $k = 0.38$ (rad/s), $\sigma/d = 0.314$ (rad/s$^{1/2}$), and varying $\Delta\omega$)], we find that as $\langle\rho\rangle$ increases from 0.3 (light grey) to 0.4 (dark grey) and finally, to 0.5 (black), the motion becomes less uniform and approaches slow-fast dynamics.

IV. HYDRODYNAMIC MODEL FOR SYNCHRONIZATION

Swimming micro-organisms are generally modelled as force dipoles [13]. For an *E.coli* cell, the flagella produce a thrust that is responsible for locomotion and the cell body exerts a force in the opposite direction. Since only the flagella produce thrust but the entire cell experiences drag, the centres of thrust and drag are physically separated which creates a dipolar fluid flow. Consider a cell moving in a straight line, shown in Figure S9a. The force is $\mathbf{F} = F\mathbf{e}$, where F is the magnitude, which is assumed to be constant, and $\mathbf{e}$ is the direction of the force and the line joining the centres of drag and thrust is given by $\epsilon l\mathbf{d}$, where ϵ is a small dimensionless number and $\mathbf{d}$ is the direction. For a cell swimming in a straight line, the vectors $\mathbf{e}$ and $\mathbf{d}$ are identical and the resulting force dipole is a *stresslet*.

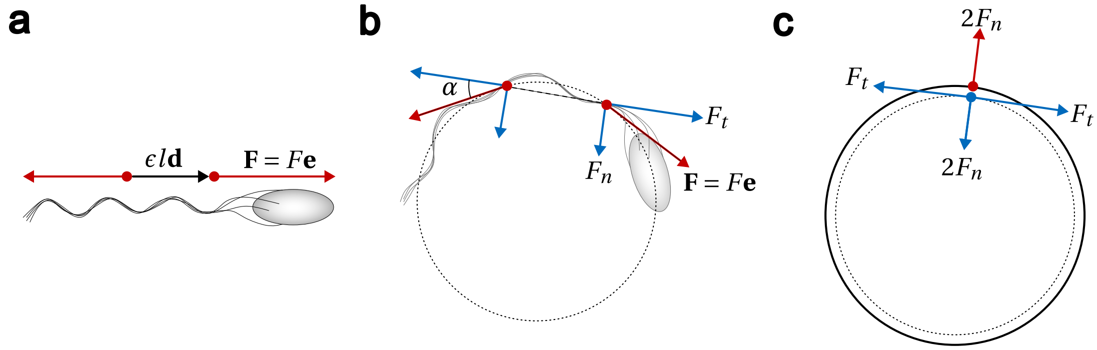

Fig S9: (a) *E.coli* as a force dipole. (b) Force decomposition when an *E.coli* cell is moving on a circular track (c) The effect of the cavity wall balancing the normal force

However, the situation is different when an *E.coli* cell is swimming in a circular trajectory, such as in a microcavity. As shown in Figure S9b, due to the curvature of the microcavity, the thrust and drag forces are decomposed into tangential and normal components relative to the motion of the cell with strengths F_t and F_n respectively. Since ϵ is small, the tangential component acts as a stresslet with dipole strength $\epsilon l F_t$ and the normal component acts as a *stokeslet* of strength $2F_n$. However, motion of micro-organisms at low Reynolds numbers is force-free and torque-free, meaning that the normal force is balanced by the walls of the circular microcavity that the cells are swimming inside. This implies that the presence of a channel is a straight forward way to induce synchronization, by means of a net force being exerted on the fluid. Also, for a bead moving on a periodic trajectory, it has been shown that a constant tangential force cannot lead to synchronization [14]. This argument can be extended to a force dipole which means that the tangential stresslet component does not contribute to the synchronization of *E.coli* oscillators.

Now, consider a pair of cells in adjacent cavities connected by a microchannel as shown in Figure S7. Each cell has a local normal vector $\mathbf{n}_i = (\cos\phi_i, \sin\phi_i)$ and tangential vector $\mathbf{t}_i = (\sin\phi_i, -\cos\phi_i)$ where $i = 1, 2$. Since the cells are forced to move on a circular trajectory, the velocity of each cell can be written as $\mathbf{v}_i = \frac{d}{2}\dot{\phi}_i\mathbf{t}_i$. This velocity is the sum of self interaction of the cell and interaction due to the other cell. The self interaction is the velocity generated due to flagellar thrust given by $\frac{d}{2}\omega_i\mathbf{t}_i$ and involves complicated mobility relations for the cell head, flagella and walls of the cavity but it can be measured from experiments as shown in Table II from which the rotary frequency ω_i can be obtained (See main text). The interaction due to the other cell is $\mathbf{G}(\mathbf{r}) \cdot \mathbf{F}$ where $\mathbf{G}$ is the Oseen tensor that gives the flow velocity at a distance $\mathbf{r}$ due to a force $\mathbf{F}$. The velocity of each cell can thus be written as,

$$\frac{d}{2}\dot{\phi}_1 = \frac{d}{2}\omega_1 + \mathbf{t}_1 \cdot \mathbf{G} \cdot \mathbf{F}_2 \quad (3)$$

$$\frac{d}{2}\dot{\phi}_2 = \frac{d}{2}\omega_2 + \mathbf{t}_2 \cdot \mathbf{G} \cdot \mathbf{F}_1 \quad (4)$$

where $\mathbf{F}_{1,2} = -2F_n\mathbf{n}_{1,2}$. The negative sign is because the normal force is pointed into the cavity. For a cell in a $7\mu m$ cavity, the force is at an angle $\alpha \approx 30°$ with respect to the local tangent giving $\mathbf{F}_{1,2} = -F\mathbf{n}_{1,2}$. $\mathbf{G}$ is the same for both cells as the distance vector is unchanged when both cells are interchanged. A dot product is taken with the tangential vector since only the tangential velocity component is responsible for the circular motion.

The action of the stokeslet in the channel can be approximated by the action of a stokeslet between two parallel flat plates. In the far field, this flow field, defined by $\mathbf{v} = \mathbf{G} \cdot \mathbf{D}$, acts as a 2 dimensional source dipole of strength $\mathbf{D}$ for a unit force [15]. $\mathbf{D}$ is a vector that gives the orientation of the source dipole and in this case, it is along the direction of $\mathbf{F}_{1,2}$. The Oseen tensor is then given by

$$\mathbf{G} = \frac{1}{2\pi}\left(-\frac{\mathbf{I}}{r^2} + \frac{2\mathbf{rr}}{r^4}\right) \quad (5)$$

where $\mathbf{I}$ is the Identity tensor (dimension 2) , $\mathbf{r} = (d + c_l + \frac{1}{2}d(\cos\phi_2 - \cos\phi_1), \frac{1}{2}d(\sin\phi_2 - \sin\phi_1))$ is the distance vector between the cells, $\mathbf{rr}$ is a dyadic product and $r = |\mathbf{r}|$. For a force with strength F near a wall at a distance $c_w/2$, the strength of the source dipole in the far field is $c_w^2 F/2$ [16]. Assuming that the action of the stokeslet is always in the centre of the channel, the phases of the oscillators can be given by

$$\dot{\phi}_1 = \omega_1 - \frac{3c_w F}{16\pi\mu d}\mathbf{t}_1 \cdot \left(-\frac{\mathbf{I}}{r^2} + \frac{2\mathbf{rr}}{r^4}\right) \cdot \mathbf{n}_2 \quad (6)$$

$$\dot{\phi}_2 = \omega_2 - \frac{3c_w F}{16\pi\mu d}\mathbf{t}_2 \cdot \left(-\frac{\mathbf{I}}{r^2} + \frac{2\mathbf{rr}}{r^4}\right) \cdot \mathbf{n}_1 \quad (7)$$

where μ is the viscosity of the fluid and the normal vector simplifies to $\mathbf{n}_i = (\cos\phi_i, 0)$ as force components perpendicular to the channel decay exponentially [15]. To simplify this equation into a form similar to the Adler model, consider a state close to synchronization where $\phi_1 \approx \phi_2$. The distance vector becomes $\mathbf{r} = (d + c_\ell, 0)$. Defining the phase difference as $\varphi = \phi_2 - \phi_1$ and taking the difference of Equations 7 and 6,

$$\dot{\varphi} = \Delta\omega - \frac{3c_w F}{16\pi\mu d(d + c_\ell)^2}\sin\varphi \tag{8}$$

Comparing this to the Adler equation, we obtain a coupling value k in terms of physical parameters of the system. In particular, k appears to be proportional to the channel width but inversely proportional to the square of the distance between the cells (and indirectly the channel length). For a $7\mu m$ cavity and a thrust force of $F = 1pN$, the coupling values are shown in Table III. The coupling values obtained here are consistent and comparable with the values extracted from the probability distributions of phase difference obtained from experiments (See Figure 3 in the main text). The difference in values could have multiple origins like the simplistic assumptions behind the toy model and practical considerations such as imperfections in the rotary motion due to the PDMS microcavity.

k (rad/s)	$c_\ell = 0.5$ μm	$c_\ell = 1$ μm	$c_\ell = 2$ μm
$c_w = 0.5$ μm	0.08	0.07	0.05
$c_w = 0.7$ μm	0.12	0.09	0.07
$c_w = 0.9$ μm	0.14	0.12	0.1

TABLE III: The coupling parameter k as a function of the channel width and length from the hydrodynamic model.

V. EXTENDED DATA

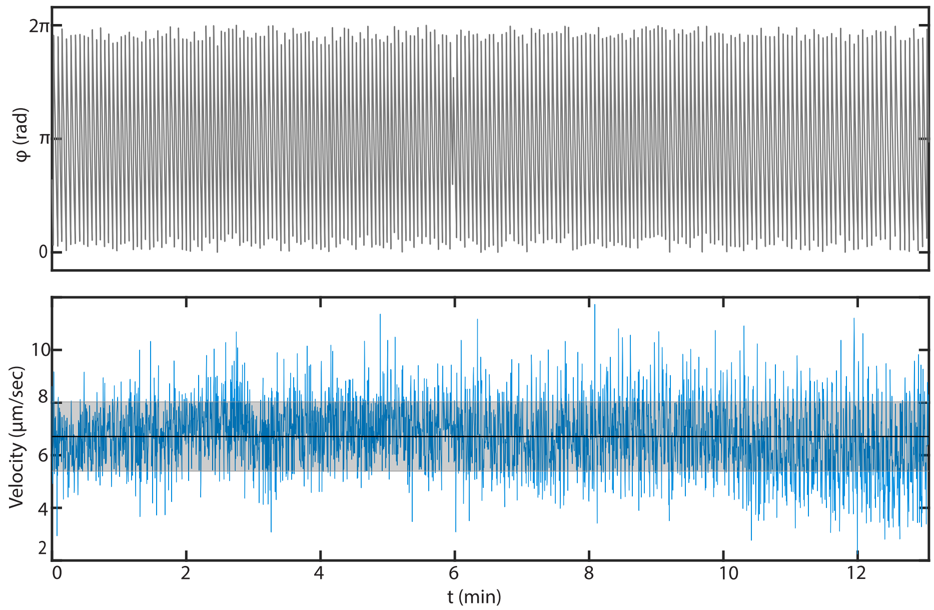

Fig S10: Measurement of cell motion (relative to the cavity center) inside an $8\mu m$ microcavity. The top panel displays cell phase angle, and the bottom panel cell velocity, with the mean speed (black line) and the standard deviation (grey shade). Trapping was sustained for over 13min, during which the bacterium was observed to traverse the cavity, performing clockwise rotations.

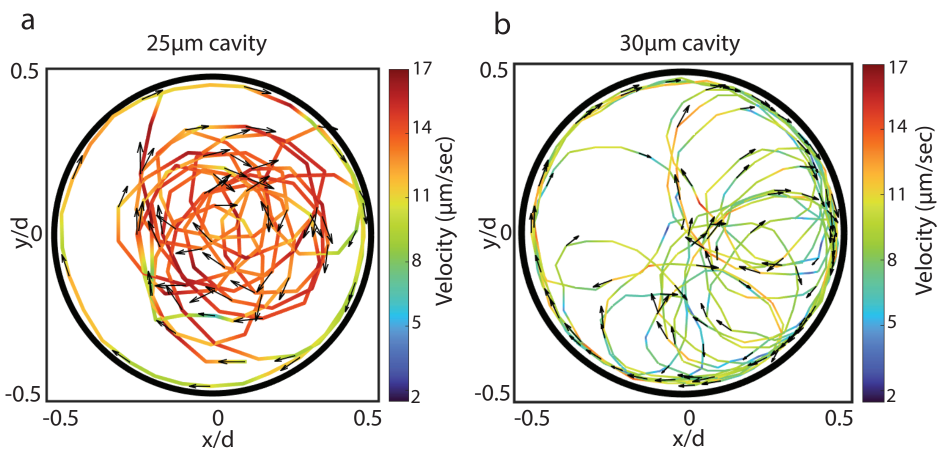

Fig S11: Cell trajectories of trapped *E. coli* in large cavities. The position and velocity maps of the single-cell are displayed for (a) 25μm cavity, and (b) 30μm cavity. The tracking of the cell inside the cavity was conducted with ImageJ software.

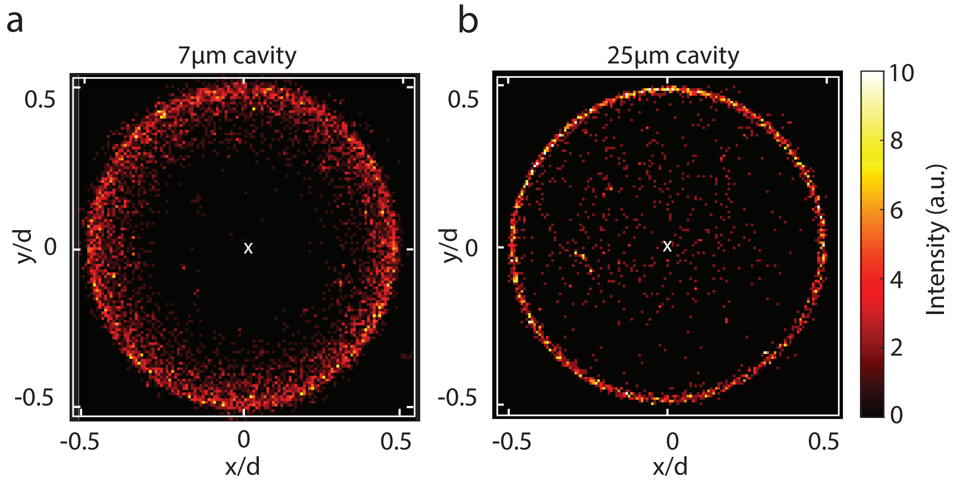

Fig S12: Distribution of *E. coli* motion in confinement. (a) Averaged heat map of *E. coli* motion in 7μm microwell from $N = 226$ measurements, (b) Averaged heat map of *E. coli* motion in 25μm microwell, from $N = 100$ measurements.

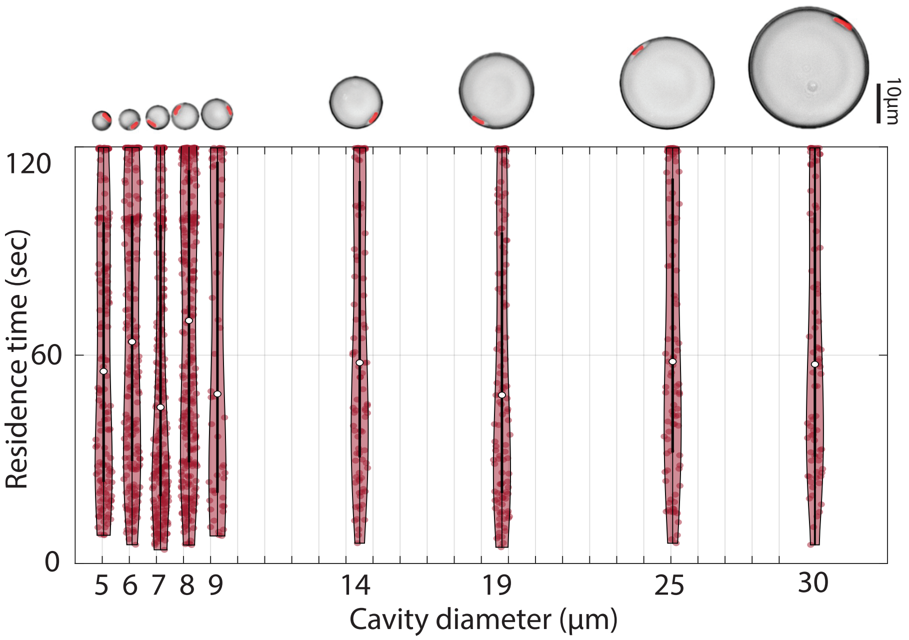

Fig S13: Residence time distribution of *E. coli* in confinement. The trapping time appears to be independent of the microcavity diameter. The recordings were cut-off at the $2min$ mark. The number of measurements per microcavity is as in Fig. 2.

VI. SUPPLEMENTARY VIDEOS

Video S1. Trapping *E. coli* cells with single-cell resolution over an array of PDMS microcavities ($d = 8\ \mu m$).
Video S2. Self-sustained oscillations of a single *E. coli* cell inside a circular microcavity ($d = 8\ \mu m$).
Video S3. Self-sustained oscillations of a single *E. coli* cell inside a rectangular (6x9 μm^2) microcavity.
Video S4. Clockwise motion of a single *E. coli* cell inside a square labyrinth having 30 μm long and 4 μm wide microchannels.
Video S5. Periodic motion of a single *E. coli* inside inverted circular microcavities ($d = 30\ \mu m$).
Video S6. Motion of a single *E. coli* inside large microcavities ($d = 25\ \mu m$) showcasing two distinct dynamics, namely spiraling and periodic wall-following.
Video S7. Uncoupled motion of a pair of *E. coli* cells in adjacent microcavities ($d = 8\ \mu m$).
Video S8. Coupled dynamics and phase-slips in a pair of *E. coli* cells moving in microcavities ($d = 8\ \mu m$) and connected by microchannels (c_w=0.5 μm and c_l=0.5 μm).
Video S9. Synchronization of a pair of *E. coli* cells moving in microcavities ($d = 7\ \mu m$) and connected by microchannels (c_w=0.5 μm and c_l=0.5 μm).
Video S10. Synchronous motion of multiple *E. coli* cells in an asymmetric doublet with a square and a circular side connected by a 4 μm channel. The doublet was designed to concentrate cells on the left side.

REFERENCES

[1] DePalma. V., Tillman, N., Friction and Wear of Self -Assembled Trichlorosilane Monolayer Films on Silicon, Langmuir (1989).
[2] Kim, G., Lee, S., Kim, C., Assessment of the Physical, Mechanical, and Tribological Properties of PDMS Thin Films Based on Different Curing Conditions, Materials (2021).
[3] Merouane, A., Rey-Villamizar, N. et al, Automated profiling of individual cell-cell interactions from high-throughput time-lapse imaging microscopy in nanowell grids (TIMING), Bioinformatics (2015).

[4] Xie, J., Khan, S., Shah, M., Automatic tracking of escherichia coli in phase-contrast microscopy video, IEEE Transactions on Biomedical Engineering (2009).
[5] Van Valen, D., Kudo, T., Lane, K. et al., Deep Learning Automates the Quantitative Analysis of Individual Cells in Live-Cell Imaging Experiments, PLoS computational biology (2016).
[6] Lugagne, J., Lin, H., Dunlop, M., DeLTA: Automated cell segmentation, tracking, and lineage reconstruction using deep learning, PLoS computational biology (2020).
[7] Yuen, H., Princen, J. et al, Comparative study of Hough Transform methods for circle finding, Image and Vision Computing (1990).
[8] Adler, R., A Study of Locking Phenomena in Oscillators, Proceedings of the IRE (1946).
[9] Bhansali, P., Roychowdhury, J., Gen-Adler: the Generalized Adler's equation for injection locking analysis in oscillators, ASP-DAC '09: Asia and South Pacific Design Automation Conference (2009).
[10] Oliaei, O., Synchronization and phase synthesis using PLL neural networks, IEEE International Symposium on Circuits and Systems (2006).
[11] Liebchen, B., Cates, M., Marenduzzoa, D. , Pattern formation in chemically interacting active rotors with self-propulsion, Soft Matter (2016).
[12] M. Scheffer, J. Bascompte, W. A. Brock, V. Brovkin, S. R. Carpenter, V. Dakos, H. Held, E. H. Van Nes, M. Rietkerk, and G. Sugihara, Early-warning signals for critical transitions, Nature **461**, 53 (2009).
[13] E. Lauga, *The Fluid Dynamics of Cell Motility*, Cambridge Texts in Applied Mathematics (Cambridge University Press, 2020).
[14] N. Uchida and R. Golestanian, Generic conditions for hydrodynamic synchronization, Physical Review Letters **106**, 10.1103/PhysRevLett.106.058104 (2011).
[15] N. Liron and S. Mochon, Stokes flow for a stokeslet between two parallel flat plates, Journal of Engineering Mathematics **10**, 10.1007/BF01535565 (1976).
[16] J. R. Blake, A note on the image system for a stokeslet in a no-slip boundary, Mathematical Proceedings of the Cambridge Philosophical Society **70**, 10.1017/S0305004100049902 (1971).
